# Antithrombotic therapy strategies for atrial fibrillation patients undergoing percutaneous coronary intervention: A systematic review and network meta-analysis

**DOI:** 10.1371/journal.pone.0186449

**Published:** 2017-10-12

**Authors:** Xiaoxuan Gong, Shaowen Tang, Jiangjin Li, Xiwen Zhang, Xiaoyi Tian, Shuren Ma

**Affiliations:** 1 Department of Cardiology, Huai’an First People’s Hospital, Nanjing Medical University, Huai’an, Jiangsu, People’s Republic of China; 2 Department of Epidemiology, School of Public Health, Nanjing Medical University, Nanjing, Jiangsu, People’s Republic of China; University of Bologna, ITALY

## Abstract

**Objective:**

The aim of this systematic review and network meta-analysis was to evaluate the comparative efficacy and safety of antiplatelet agents, vitamin K antagonist (VKA) and non-VKA oral anticoagulants (NOACs) in patients with atrial fibrillation (AF) undergoing percutaneous coronary intervention (PCI).

**Methods:**

PubMed, EMBASE, and the Cochrane Central Register of Controlled Trials were searched to identify clinical trials comparing antiplatelet drugs with VKA and NOACs or their combination in AF patients undergoing PCI with a mean/median follow-up of at least 12 months. A network meta-analysis was conducted to directly and indirectly compare the efficacy and safety of competitive antithrombotic regimens with a Bayesian random-effects model. Results were presented as relative risks (RRs) and 95% confidence intervals (CIs).

**Results:**

A total of 15 studies enrolling 13,104 patients were included. Among 5 regimens, rivaroxaban 15 mg daily plus P2Y_12_ inhibitor treatment demonstrated significant superiority over dual- and triple-antiplatelet therapies (DAPT, TT) in reducing thromboembolic events (0.64 [0.38, 0.95] and 0.68 [0.43, 0.98], respectively) but showed the maximum possibility of major bleeding risk, while VKA plus single antiplatelet therapy (SAPT) seemed the safest. Significantly less risk of major bleeding was seen in DAPT group than that in TT group (0.63 [0.39, 0.99]).

**Conclusions:**

The present study suggests that combination of VKA and SAPT is the best choice for AF patients undergoing PCI considering both efficacy and safety. Rivaroxaban 2.5 mg twice daily plus DAPT treatment owns the highest probability to be the optimal alternative to VKA plus SAPT for these patients.

## Introduction

Percutaneous coronary intervention (PCI) with dual-antiplatelet therapy (DAPT, aspirin and P2Y_12_ inhibitor or thienopyridine) is a standardized treatment for coronary artery disease (CAD) patients with moderate or severe coronary artery stenosis, 5% to 15% of whom are concomitant atrial fibrillation (AF)[1–3]. A previous observational study reported that 12% of 113,283 PCI cases were concomitant AF and significantly older, more likely to have comorbid congestive heart failure, cardiomyopathy, cerebrovascular disease, chronic lung disease and in-hospital complications, including in-hospital mortality (3.2% *vs*. 1.3%, *p* < 0.001)[4]. Anticoagulant treatment is of great importance to prevent thrombosis in AF patients while DAPT owns Ia evidence after PCI. As to the antithrombotic therapy for AF patients undergoing elective PCI, 2016 European Society of Cardiology (ESC) guidelines recommend triple therapy (TT) including oral anticoagulant (OAC), aspirin 75–100 mg per day and clopidogrel 75 mg per day for a period of 1 months (evidence level IIaB), followed by dual therapy (OAC with aspirin or clopidogrel, evidence level IIaC) till 6 or 12 months according to high or low bleeding risk and then OAC for lifelong (evidence level IB)[5]. However, the clinical evidence by now has been deficient to support the strategy decision and prescribe individualized antithrombotic regimens for AF patients undergoing PCI.

Notably, administration of VKA requires frequent monitoring of coagulant function and adjustment for proper dosage because of its fluctuated pharmacokinetics and susceptibility to food and drugs. Conversely, non-VKA oral anticoagulant (NOAC) without these deficiencies has dramatically optimized anticoagulation management. Although antithrombotic efficacy and bleeding risk of NOACs for AF patients undergoing PCI in the real world has not been well established yet, several clinical studies have yielded different results of several antiplatelet and anticoagulant regimens for these patients[6–20]. This systematic review and network meta-analysis (NMA) aimed to rank efficacy and safety of different antithrombotic therapies available for AF patients in post-PCI.

## Methods

### Systematic review

A systematic review protocol was prepared to define all aspects of the review beforehand (https://dx.doi.org/10.17504/protocols.io.iijcccn). All the participants, interventions, comparisons, outcomes, and study designs (PICOS) were recorded in the systematic review process.

### Search strategy and data sources

We conducted a systematic literature search in PubMed, Embase and Cochrane Central databases from the earliest possible search date through March 2017. The following search terms were used: (“atrial fibrillation” or “AF”) and (“percutaneous coronary intervention” or “PCI” or “coronary stent implantation”) and (“aspirin” or “P2Y_12_ antagonist” or “P2Y_12_ inhibitor” or “clopidogrel” or “ticagrelor” or “warfarin” or “vitamin K antagonist” or “VKA” or “dabigatran” or “rivaroxaban” or “apixaban” or “edoxaban” or “novel oral anticoagulant” or “new oral anticoagulant” or “NOAC”).

### Study selection and eligibility criteria

Two reviewers (GXX and TSW) performed the literature selection, data extraction, and quality assessment. Disagreements were resolved by the consensus of a third reviewer (LCJ). The study selection process involved literature screening through titles and/or abstracts and further full-text evaluation.

The inclusion criteria were as follows: (1) clinical trials with at least one compared group; (2) study population of CAD patients with paroxysmal, persistent, or permanent AF undergoing PCI; (3) treatment with antiplatelet or anticoagulant agents mentioned before with a target international normalized ratio (INR) of 2.0–3.0 when warfarin was used to reflect present standard practice; (4) outcomes involving main adverse cardiac and cerebrovascular events (MACCEs) and major bleeding; (5) mean or median follow-up period no less than 12 months. Studies were excluded if (1) they were published only in the form of abstracts or in non-English languages; (2) antithrombotic therapies changed differently in post-PCI; (3) compared treatments were not specific such as VKA *vs*. non-VAK.

Duplicates were firstly and irrelevant or non-English articles were subsequently removed on the basis of titles and/or abstracts. After assessment of full-text studies, eligible ones were finally enrolled in our NMA.

### Data extraction and quality assessment

The extracted data mainly included study design, sample size (all and in compared arms), follow-up duration, antithrombotic strategies in post-PCI, efficacy and safety outcomes, baseline characteristics of the patients including mean age, male percentage, mean CHADS_2_/CHA_2_DS_2_-VASc and HAS-BLED scores and most importantly the number and percentage of MACCEs and major bleeding outcomes in every arm.

The qualities of observational studies and randomized controlled trials (RCTs) were assessed by the Newcastle-Ottawa Scale (NOS, evaluation of selection, comparability and outcome)[21] and Jadad Scale (judgment of randomization, double blind, withdrawals and dropouts)[22] respectively. As the Cochrane Collaboration recommended[23], all qualified studies were included in our analysis without considering their qualities.

### Outcome measures

The primary endpoint was defined as the occurrence of MACCEs, including all-cause mortality, cardiac death, myocardial infarction (MI), target lesion revascularization (TLR), and stroke. The secondary outcome was major bleeding, defined by TIMI[24] or the Bleeding Academic Research Consortium (BARC)[25] criteria. The endpoints data in around 1-year follow-up available were collected for our analysis.

### Statistical analysis

Results for endpoint events were treated as dichotomous data. Risk ratio (RR) and 95% confidence interval (CI) were used to estimate pooled results from studies. A Bayesian NMA was conducted in Addis 1.16.8 software by Markov chain Monte Carlo methods, which provided direct and indirect evidence for any given treatments in one joint analysis and a possible ranking distribution of 2 endpoints for all regimens[26]. If 95% CI of inconsistency factors median contained 0 and *p* value of node split model was over 0.05, inconsistent model would be considered not significant and then consistent model would be used for further analysis.

## Results

### Search results and studies characteristics

**Fig 1:**
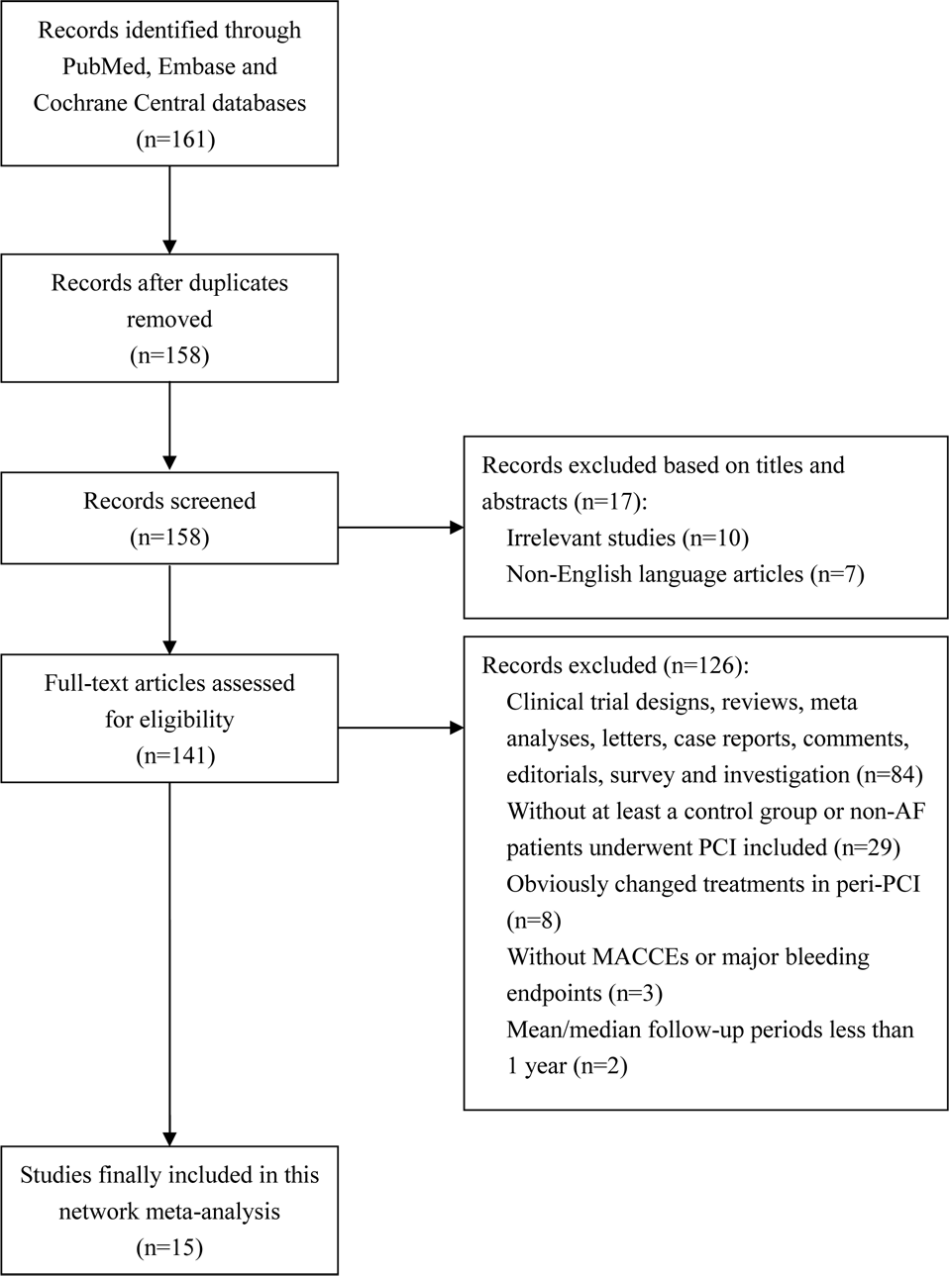
Flow diagram of study selection in this net-work meta-analysis.

A total of 161 potentially relevant titles were identified by the key words mentioned before. After 146 duplicate analysis (n = 3), non-English articles (n = 7), irrelevant studies (n = 10), non-clinical trials (n = 84), and clinical trials not fulfilling inclusion/exclusion criteria (n = 42) were rejected, 15 studies including 13,104 patients were finally included in this NMA after screening and full-text assessment (Fig 1). According to the principles expressed in the Declaration of Helsinki, all participants provided written consents in every enrolled trial.

The studies and patients baseline characteristics are shown in Table 1. We incorporated 3 RCTs and 12 observational cohort studies with a mainly mean or median follow-up of 1 year using 5 regimens: DAPT (n = 6,489), TT (n = 4,721), rivaroxaban 15 mg per day plus P2Y_12_ inhibitor (n = 757), rivaroxaban 2.5 mg twice daily plus DAPT (n = 706) and VKA plus single antiplatelet therapy (SAPT) (n = 434). INR therapeutic range was 2.0–3.0 when VKA was used. Loading doses of aspirin and ticagrelor or clopidogrel were administered to acute coronary syndrome (ACS) patients and before PCI according to guideline[27]. Among the recruited patients, the reported mean age was similar across trials (68.3–79.6 years) and men were in the majority (45%-82.4%). The mean or median values of 12 CHADS_2_/CHA_2_DS_2_-VASc scores and 7 HAS-BLED scores were mostly over 2 which indicated high risks of both thrombosis and bleeding in enrolled patients. All of the included studies reported thromboembolic events and major bleeding endpoints. The rates of cardiovascular-cause mortality and re-hospitalization were used for MACCEs outcome evaluation of PIONEER AF-PCI trial[19].

**Table 1:** Main characteristics of studies and patients included in the network meta-analysis.

Studies characteristics					Patients baseline characteristics			
Trial name/country, author, published year	Study design	Sample size	Follow-up period	Treatment groups	Age (years)	Male (%)	CHADS_2_/ CHA_2_DS_2_-VASc score^3^	HAS-BLED score^4^
Germany, Lars Maegdefessel, et al, 2008^[^^6^^]^	Single-center, prospective cohort trial	117	1.4 years	DAPT, TT	69.6	74.4	-	-
WOEST, Willem J M Dewilde, et al, 2013^[^^7^^]^	Multi-center, randomized, open-label, controlled trial	573	1 year	TT, VKA + clopidgrel^1^	68.7	78.2	≥2, 49.7%	-
Poland, Magdalena Dąbrowska, et al, 2013^[^^8^^]^	Single-center, prospective cohort trial	104	1 year	DAPT, TT	70.2	58.7	-	-
CRUSADE, Emil L. Fosbol, et al, 2013^[^^9^^]^	Multi-center, retrospective cohort trial	1648	1 year	DAPT, TT	77.7	58.3	2/4	-
Japan, Hideki Kawai, et al, 2014^[^^10^^]^	Multi-center, retrospective cohort trial	146	37 months	DAPT, TT, VKA + SAPT	72.0 ± 8.1	72.9	2.14	≥3, 32.9%
AFCAS, Andrea Rubboli, et al, 2014^[^^11^^]^	Multi-center, prospective cohort trial	914	1 year	TT, DAPT, VKA + clopidgrel^1^	73 ± 8	70	2.2 ± 1.2	3.0 ± 0.7
Korea, Soon Yong Suh, et al, 2014^[^^12^^]^	Single-center, retrospective cohort trial	203	42.0 ± 29.0 months	DAPT, TT	68.3 ± 10.1	62.6	1.92 ± 1.19	1.97 ± 0.64
Korea, Dong Oh Kang, et al, 2015^[^^13^^]^	Two-center, retrospective cohort trial	367	2 years	DAPT, TT	68.1	65.1	1.82	-
ACTION Registry–GWTG, Connie N. Hess, et al, 2015^[^^14^^]^	Multi-center, prospective cohort trial	4959	2 years	DAPT, TT	77.5	57.5	2.54	-
AVIATOR, Marco G. Mennuni, et al, 2015^[^^15^^]^	Multi-center, prospective cohort trial	859	1 year	DAPT, TT	73 ± 9.6	71	2.7 ± 1.2	2.9 ± 0.7
Span, Antonia Sambola, et al, 2016^[^^16^^]^	Multi-center, prospective cohort trial	585	1 year	DAPT, TT	73.2 ± 8.2	75.2	≥2, 73.2%	≥3, 39.2%
Triple Therapy in Elderly Patients, Antonia Sambola, et al, 2016^[^^17^^]^	Multi-center, prospective cohort trial	289	1 year	DAPT, TT	79.6 ± 3.4	67.1	4.0 ± 1.4	≥3, 84.4
ROCKET AF, Matthew W. Sherwood, et al, 2016^[^^18^^]^	Multi-center, randomized, double-blind, double-dummy noninferiority controlled trial	153	806 days	TT, rivaroxaban^2^ + P2Y_12_ inhibitor	73 (67, 79)	82.4	3.5 ± 1.0	-
PIONEER AF-PCI, Gibson CM, et al, 2016^[^^19^^]^	Multi-center, randomized, open-label, controlled trial	2124	1 year	TT, rivaroxaban 15 mg/d + P2Y_12_ inhibitor; rivaroxaban 2.5 mg bid + DAPT	70.1	74.4	3.77	-
Italy, Renato De Vecchis, et al, 2016^[^^20^^]^	Single-center, retrospective cohort trial	98	378 ± 15.7 days	DAPT, TT, VKA + SAPT	73 ± 7.5	45	1.8 ± 1.5/5.3 ± 1.6	2.3 ± 0.5

1. Clopidogrel 75 mg/d

2. Rivaroxaban 20 mg/d or 15 mg/d in participants with a creatinine clearance of 30–90 ml/min

3. 1 point each for the presence of congestive heart failure, hypertension, age 75 years or older, and diabetes mellitus, and 2 points for history of stroke or transient ischemic attack

4. 1 point each for hypertension, abnormal renal and liver function, stroke, bleeding, labile INRs, age at least 65 years, drugs or alcohol. Results were shown as mean ± standard deviation (SD) or median value with or without interquartile range.

Abbreviations: VKA: vitamin K antagonist; DAPT: dual antiplatelet therapy including aspirin 75–100 mg/d, clopidogrel 75 mg/d; TT: triple therapy including aspirin 75–100 mg/d, clopidogrel 75 mg/d and VKA; SAPT: single antiplatelet therapy, aspirin 75–100 mg/d or clopidogrel 75 mg/d.

### Individual studies results

The raw data of this NMA are presented in S1 Table. All of 15 enrolled trials reported TT outcomes and 13 demonstrated DAPT endpoints. The remaining 3 regimens including VKA plus SAPT, rivaroxaban 15 mg per day plus P2Y_12_ inhibitor and rivaroxaban 2.5 mg twice daily plus DAPT were observed in 4, 2 and 1 trials respectively. MACCEs incidence rates varied from 3.3% in DAPT arm[8] to 37.3% in VKA with SAPT arm[10], and major bleeding rates were observed from 0[6] to 21.4%[10] both in TT group.

### NMA results

**Fig 2:**
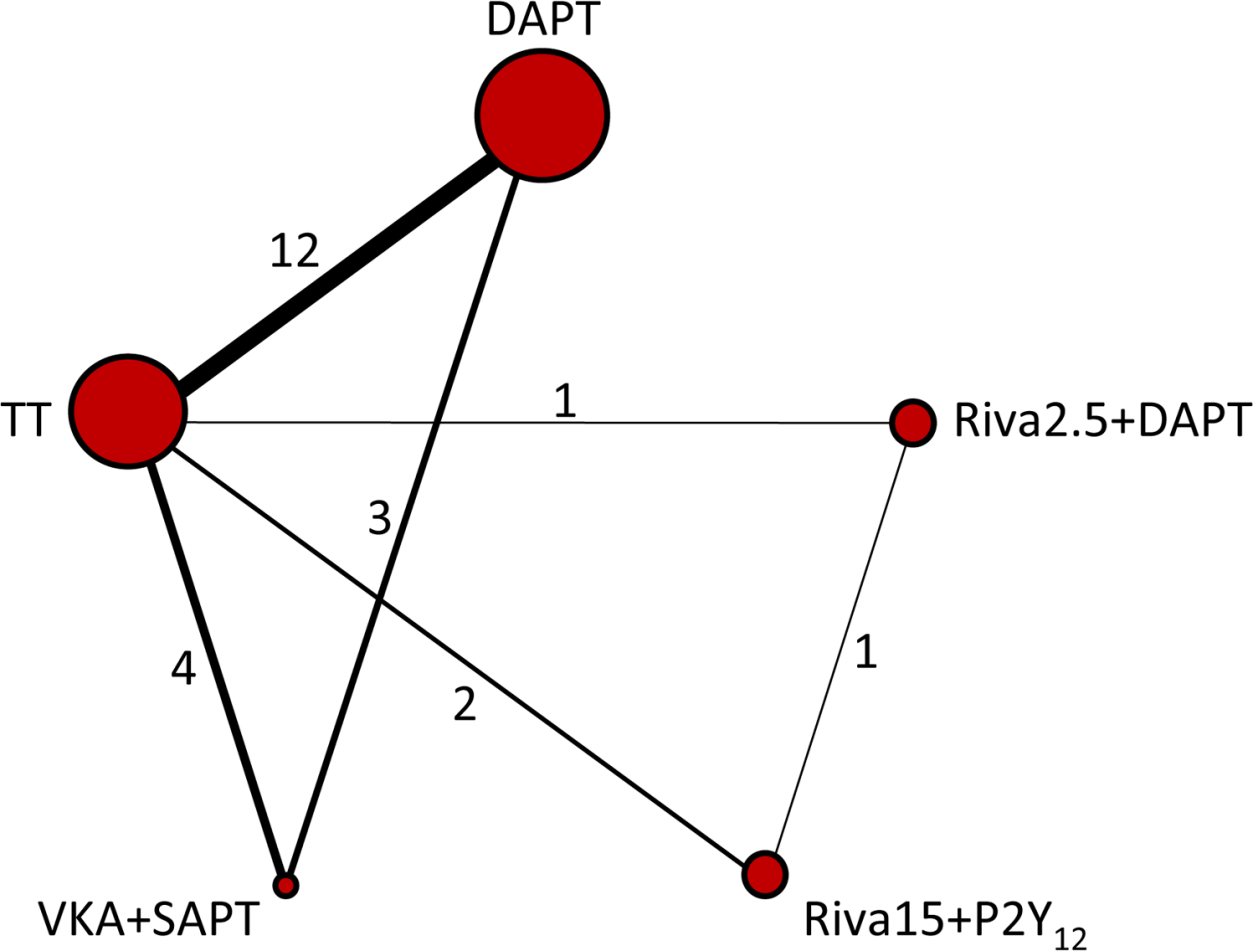
Network diagram of treatment comparisons for MACCEs and major bleeding of competitive antithrombotic regimens. MACCEs, main adverse cardiac and cerebrovascular events; DAPT, dual-antiplatelet therapy; TT, triple-antiplatelet therapy; VKA, vitamin K antagonist; SAPT, single antiplatelet therapy; Riva15 + P2Y_12_, rivaroxaban 15 mg/d plus P2Y_12_ inhibitor; Riva2.5 + P2Y_12_, rivaroxaban 2.5 mg bid plus P2Y_12_ inhibitor. The size of each node is proportional to the overall sample size of the corresponding therapy. Each line represents the direct comparison between two treatments, and the corresponding width is proportional to the number of trials. Number next to the line indicates the specific number of studies.

**Fig 3:**
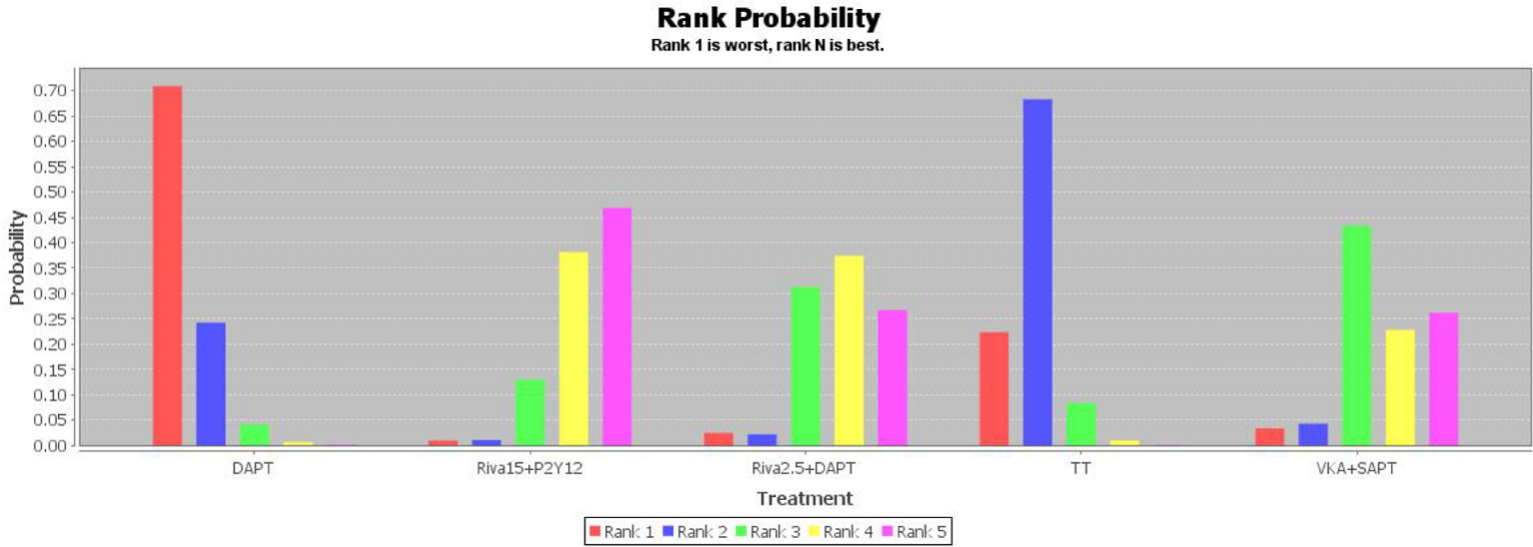
The distribution of MACCEs probabilities of 5 treatments being ranked at possible positions. MACCEs, main adverse cardiac and cerebrovascular events; DAPT, dual-antiplatelet therapy; Riva15 + P2Y_12_, rivaroxaban 15 mg/d plus P2Y_12_ inhibitor; Riva2.5 + P2Y_12_, rivaroxaban 2.5 mg bid plus P2Y_12_ inhibitor; TT, triple-antiplatelet therapy; VKA, vitamin K antagonist; SAPT, single antiplatelet therapy.

**Fig 4:**
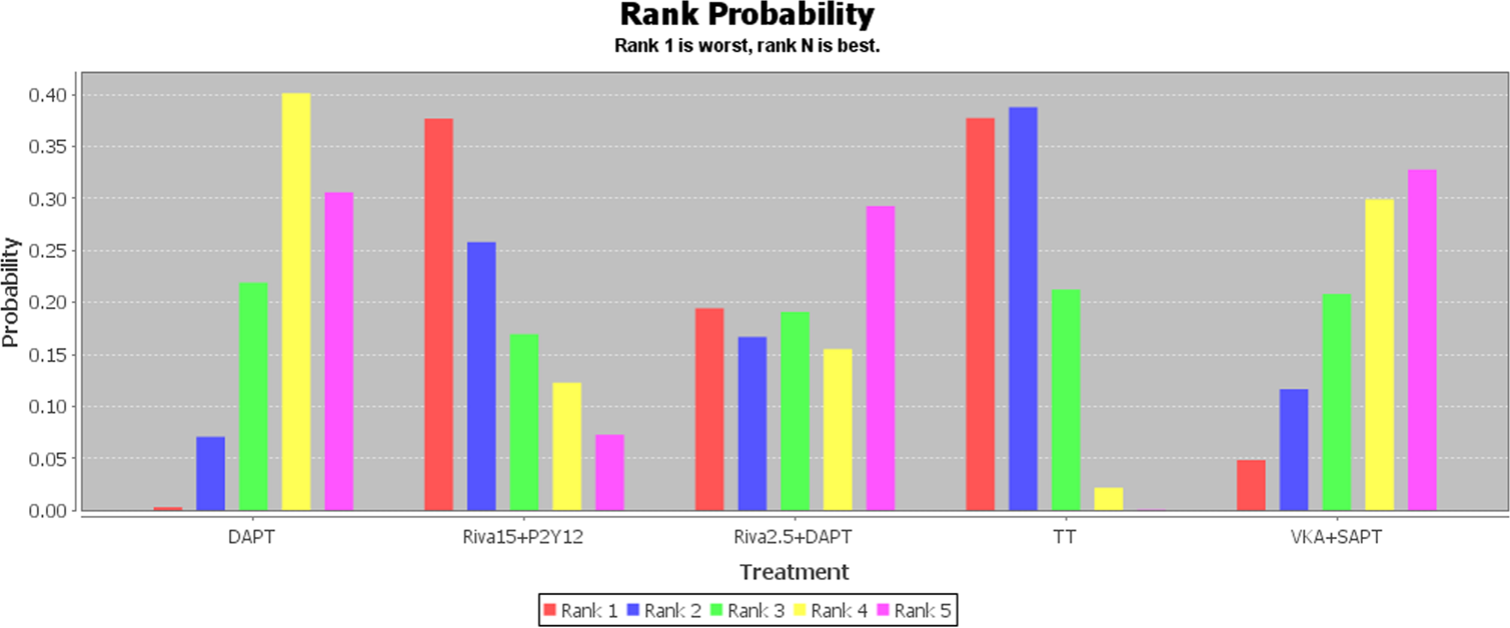
The distribution of major bleeding probabilities of 5 treatments being ranked at possible positions. DAPT, dual-antiplatelet therapy; Riva15 + P2Y_12_, rivaroxaban 15 mg/d plus P2Y_12_ inhibitor; Riva2.5 + P2Y_12_, rivaroxaban 2.5 mg bid plus P2Y_12_ inhibitor; TT, triple-antiplatelet therapy; VKA, vitamin K antagonist; SAPT, single antiplatelet therapy.

Network of evidence for MACCEs and major bleeding outcomes was displayed in Fig 2. In all, 12 studies made a comparison between TT and DAPT, followed by 4 and 3 trials comparing TT and DAPT with VKA plus SAPT respectively. The distributions of two outcomes probabilities of every treatment being ranked at each of the possible 5 positions were demonstrated in Figs 3 and 4. RRs and 95% CIs for all treatments relative to each other in MACCEs and major bleeding endpoints under the consistency model were presented in Tables 2 and 3.

**Table 2:** NMA results of MACCEs risk for all treatments relative to each other under the consistency model.

**DAPT**	**0.64 (0.38, 0.95)**	0.69 (0.41, 1.03)	0.95 (0.79, 1.13)	0.75 (0.52, 1.08)
**1.56 (1.05, 2.60)**	**Riva15 + P2Y**_**12**_	1.07 (0.70, 1.68)	**1.47 (1.03, 2.35)**	1.20 (0.70, 2.16)
1.45 (0.97, 2.46)	0.94 (0.59, 1.42)	**Riva2.5 + DAPT**	1.39 (0.94, 2.25)	1.09 (0.66, 2.01)
1.05 (0.89, 1.26)	**0.68 (0.43, 0.98)**	0.72 (0.44, 1.06)	**TT**	0.80 (0.55, 1.11)
1.33 (0.92, 1.94)	0.83 (0.46, 1.43)	0.92 (0.50, 1.52)	1.25 (0.90, 1.83)	**VKA + SAPT**

Results are presented as risk ratios (95% confidence intervals). Significant results indicated in bold. MACCEs, main adverse cardiac and cerebrovascular events; DAPT, dual-antiplatelet therapy; Riva15 + P2Y_12_, rivaroxaban 15 mg/d plus P2Y_12_ inhibitor; Riva2.5 + P2Y_12_, rivaroxaban 2.5 mg bid plus P2Y_12_ inhibitor; TT, triple-antiplatelet therapy; VKA, vitamin K antagonist; SAPT, single antiplatelet therapy.

**Table 3:** NMA results of major bleeding risk for all treatments relative to each other under the consistency model.

**DAPT**	1.77 (0.49, 7.11)	1.24 (0.25, 7.19)	**1.83 (1.11, 3.28)**	1.03 (0.40, 2.68)
0.57 (0.14, 2.03)	**Riva15 + P2Y**_**12**_	0.70 (0.14, 3.49)	1.03 (0.30, 3.50)	0.58 (0.12, 2.59)
0.81 (0.14, 4.02)	1.43 (0.29, 7.09)	**Riva2.5 + DAPT**	1.49 (0.30, 7.03)	0.83 (0.13, 4.95)
**0.55 (0.31, 0.90)**	0.97 (0.29, 3.35)	0.67 (0.14, 3.32)	**TT**	0.56 (0.22, 1.37)
0.97 (0.37, 2.48)	1.72 (0.39, 8.19)	1.20 (0.20, 7.72)	1.78 (0.73, 4.58)	**VKA + SAPT**

Results are presented as risk ratios (95% confidence intervals). Significant results indicated in bold. DAPT, dual-antiplatelet therapy; Riva15 + P2Y_12_, rivaroxaban 15 mg/d plus P2Y_12_ inhibitor; Riva2.5 + P2Y_12_, rivaroxaban 2.5 mg bid plus P2Y_12_ inhibitor; TT, triple-antiplatelet therapy; VKA, vitamin K antagonist; SAPT, single antiplatelet therapy.

#### Results of NMA for MACCEs

Combination of rivaroxaban 15 mg per day and P2Y_12_ inhibitor showed a statistically lower risk of MACCEs compared with DAPT and TT regimens for AF patients undergoing PCI (0.64 [0.38, 0.95] and 0.68 [0.43, 0.98], respectively) and owned the first place of efficacy possibility among 5 therapies (Fig 3). Besides, rivaroxaban 2.5 mg twice daily with DAPT, VKA with SAPT treatments ranked in the second and third places of efficacy possibility respectively (Fig 3) were similarly superior to TT and DAPT in efficacy but with no statistical significance (Table 2). DAPT was associated with an obviously higher hazard of MACCEs than others (Fig 3).

Importantly, there were no significant inconsistencies between the direct and indirect evidence of MACCEs in all closed loops (inconsistency factors median -0.22 [-0.90, 0.29]; direct effect model, -0.18 [-0.84, 0.37], indirect effect model, -0.59 [-1.24, -0.01], overall node split models, N/A, *p* = 0.36).

#### Results of NMA for major bleeding

Regarding the outcome of major bleeding, the least risk was observed in VKA plus SAPT then DAPT treatment, whereas rivaroxaban 15 mg per day plus P2Y_12_ inhibitor and TT regimens were ranked in the first and second place of risk possibility respectively (Fig 4). Besides, DAPT was significantly safer than TT in the rate of major bleeding (0.55 [0.31, 0.90]) (Table 3).

Likewise, no significant inconsistencies between the direct and indirect evidence of major bleeding were seen in all closed loops (inconsistency factors median -0.00 [-1.28, 1.22]; direct effect model, -0.03 [-1.24, 1.18], indirect effect model, 0.02 [-1.84, 1.88], overall node split models, 0.03 [-0.91, 0.99], *p* = 0.96).

#### Results of quality assessment

As were shown in S2 and S3 Tables, the mean Jadad score of 3 RCTs was 3.67 ± 1.15 and mean NOS score of 12 cohort trials was 7.17 ± 0.58. Although there were some differences between the trials in terms of study design and patient characteristics, all included studies were judged to be of good quality.

## Discussion

Our NMA, overcoming the major limitation of routine pairwise meta-analyses, provides evidence-based grading for the efficacy and safety of available oral antiplatelet and anticoagulant therapies for AF patients undergoing PCI. Although no statistical significance was identified, combination of VKA and SAPT treatment showed a trend toward achieving the greatest beneficial effect compared to other regimens, followed by rivaroxaban 2.5 mg twice daily with DAPT. DAPT only showed the weakest antithrombotic efficacy but relative safety among 5 therapies. In addition, rivaroxaban 15 mg per day plus P2Y_12_ inhibitor revealed the highest risk of major bleeding in spite of its best efficacy. The worst results were observed in TT arm which ranked in the second place of both MACCEs and major bleeding outcomes probabilities.

So far, no other NMA on antithrombotic therapy for AF patients undergoing PCI was done. Because of the heterogeneous methodology, an early systematic review by Barry AR et al[28] including 10 cohort studies and 1 meta-analysis failed to quantify the efficacy and safety of TT compared with DAPT in patients undergoing PCI with stent implantation and with multiple indications for long-term oral anticoagulation. Evidence from small cohort studies supported the benefit of TT at reducing major adverse cardiac events (MACEs) and all-cause mortality with higher rates of bleeding. However, recruited AF patients accounted for 59% to 100% in the enrolled trials who were not really representative for all AF patients. Another meta-analysis[29] on antithrombotic therapy after PCI in patients requiring OAC treatment yielded the conclusion that TT was more efficacious than dual therapy (DAPT or OAC plus SAPT) in reducing MACE/stroke [0.76 (0.70, 0.83), *p* < 0.00001 and 0.67 (0.59, 0.75), *p* < 0.00001, respectively] and significantly increased the risk of major bleeding compared with DAPT [1.36 (1.17, 1.58), *p* < 0.0001]. In the comparison between TT and OAC plus clopidogrel, there were no differences in MACCEs and major bleeding events. In our NMA, TT was associated with significantly higher risk of major bleeding compared with DAPT [1.83 (1.11, 3.28)] which was consistent with the above two meta-analyses, while VKA with SAPT seemed better than TT in efficacy for AF patients undergoing PCI (Fig 3) despite no statistical significance was reached [0.80 (0.55, 1.11)]. The population heterogeneity may be a reason for that discrepancy. A recent meta-analysis[30] compared the clinical outcomes in 20,456 AF patients undergoing PCI and stenting who received DAPT (13,253 patients) and TT (7,203 patients) with a mean follow-up period of 15 months in 18 studies and found that TT was associated with significantly lower risk of stroke, ST, and all-cause mortality compared with DAPT (odd ratios and 95% CIs were 1.98 [1.03, 3.81], *p* = 0.04; 1.59 [1.08, 2.34], *p* = 0.02; and 1.41 [1.03, 1.94], *p* = 0.03, respectively) and a significantly higher risk of major bleeding (0.62 [0.50, 0.77], *p* < 0.0001). Only 2 or 3 traditional antithrombotic treatments without NOACs were compared in the above meta-analyses, and thus not enough evidence was available for clinical decision in this condition. However, our NMA provides a rank of 5 antithrombotic therapies including NOACs for AF patients undergoing PCI.

To guarantee the study quality, several studies were discreetly excluded in out NMA for patient heterogeneity, narrow time in therapeutic range (TTR) when warfarin was used, mean/median follow-up period less than 1 year, or unspecific comparison between treatments such as VKA *vs*. non-VKA. Besides, studies in which antithrombotic therapies changed differently after PCI were also excluded to reflect their true antithrombotic efficacy and bleeding effect. A relative meta-analysis[31] which compared uninterrupted oral anticoagulation (UAC) with VKA *vs*. interrupted oral anticoagulation (IAC) with or without bridging anticoagulation before coronary procedures found that there was no difference in MACCE or major bleeding between UAC and IAC [0.74 (0.34, 1.64), *p* = 0.46; 0.62 (0.16, 2.43), *p* = 0.49, respectively]; but as compared to IAC with bridging, there was a statistically significant MACCE risk reduction and 65% lower risk of major bleeding with UAC [0.52 (0.29, 0.95), *p* = 0.03; 0.35 (0.13, 0.92), *p* = 0.03, respectively]. Thus UAC is at least as safe as IAC, and seems to be much safer than IAC with bridging anticoagulation in patients undergoing coronary angiography with or without PCI. Furthermore, only 3 RCTs out of 15 included studies could be identified to satisfy our inclusion and exclusion criteria. Several large-size RCTs for AF patients undergoing PCI with other NOACs treatments such as REDUAL-PCI (dabigatran, NCT02164864), AUGUSTUS (apixaban, NCT02415400), and ENTRUST-AF-PCI (edoxaban, NCT02866175) are on-going or just completed and published outcomes have not been available yet[32]. More results from RCTs are needed to provide evidence of optimal antithrombotic strategies for these patients.

The optimal strategy and duration of combination antithrombotic therapy for AF patients undergoing PCI is not known, but as guidelines mentioned, the continued bleeding risk suggests a short duration[5], which is consistent with the worst results observed in TT arm in this NMA. Given the lack of evidence and the greater risk of major bleeding compared with clopidogrel, the use of prasugrel or ticagrelor as part of TT should be avoided unless there is a clear need for these agents (e.g. stent thrombosis on aspirin plus clopidogrel)[33, 34]. Rivaroxaban, a direct factor Xa inhibitor, has been reported to prevent venous thromboembolism more effectively than enoxaparin in patients undergoing orthopedic surgery[35,36]. As for anticoagulant therapy for AF, ROCKET-AF demonstrated that a daily dose of 20 mg rivaroxaban was noninferior to dose-adjusted warfarin for the prevention of stroke or systemic embolism, while major bleeding from a gastrointestinal site was more common in the rivaroxaban group (3.2% vs. 2.2%, *p* < 0.001)[37]. This may partly explain why rivaroxaban 15 mg daily with P2Y_12_ inhibitor treatment ranks the worst in safety endpoint, in spite of its best efficacy in our study. The types of stent and CAD, CHA_2_DS_2_-Vasc scores and bleeding risk should be well considered of antithrombotic strategies for these patients. Besides, patients’ compliance is vital for INR management when warfarin is used. Anyhow, NOACs instead of VKA stand for the mainstream of anticoagulant therapy in the future, and proper dosage of NOACs with combined antithrombotic regimen needs further research.

In summary, this NMA suggests that combination of VKA with SAPT is an optimized strategy for AF patients undergoing PCI. Rivaroxaban 2.5 mg twice daily with DAPT may be the best alternative for VKA plus SAPT in the treatment for these patients.

## Supporting information

S1 PRISMA NMA Checklist PRISMA NMA checklist of items to include when reporting a systematic review involving a network meta-analysis. (DOCX)

S1 Table Raw data used in NMA. Abbreviations: MACCEs, main adverse cardiac and cerebrovascular events; DAPT, dual-antiplatelet therapy; Riva15 + P2Y_12_, rivaroxaban 15 mg/d plus P2Y_12_ inhibitor; Riva2.5 + P2Y_12_, rivaroxaban 2.5 mg bid plus P2Y_12_ inhibitor; TT, triple-antiplatelet therapy; VKA, vitamin K antagonist; SAPT, single antiplatelet therapy.
(DOCX)

S2 Table Quality assessment of included RCTs (Jadad scale). Abbreviations: RCT, randomized clinical trial.
(DOCX)

S3 Table Quality assessment of included cohort trials (The Newcastle-Ottawa Scale, NOS). (DOCX)
