# Supplementary material for: Antithrombotic therapy strategies for atrial fibrillation patients undergoing percutaneous coronary intervention: A systematic review and network meta-analysis
Source: PLoS One. 2017 Oct 12;12(10):e0186449. doi: 10.1371/journal.pone.0186449 (PMC5638551; doi:10.1371/journal.pone.0186449)
Supplement: S1 PRISMA NMA Checklist — (DOCX) [file pone.0186449.s001.docx]

**PRISMA NMA Checklist of Items to Include When Reporting A Systematic Review Involving a Network Meta-analysis**

| **Section/Topic** | **Item** | **Checklist Item** | **Reported** |
| --- | --- | --- | --- |
|  | **#** |  | **on Page #** |
| **TITLE** |  |  |  |
|  |  |  |  |
| Title | 1 | Identify the report as a systematic review *incorporating a* | 1 |
|  |  | *network meta-analysis (or related form of meta-analysis).* |  |
| **ABSTRACT** |  |  |  |
| Structured | 2 | Provide a structured summary including, as applicable: | 2-3 |
| summary |  | **Background:** main objectives | 2 |
|  |  | **Methods:** data sources; study eligibility criteria, participants, | 2 |
|  |  | and interventions; study appraisal; and *synthesis methods,* |  |
|  |  | *such as network meta-analysis.* |  |
|  |  | **Results:** number of studies and participants identified; | 2 |
|  |  | summary estimates with corresponding confidence/credible |  |
|  |  | intervals; *treatment rankings may also be discussed. Authors* |  |
|  |  | *may choose to summarize pairwise comparisons against a* |  |
|  |  | *chosen treatment included in their analyses for brevity.* |  |
|  |  | **Discussion/Conclusions:** limitations; conclusions and | 3 |
|  |  | implications of findings. |  |
|  |  | **Other:** primary source of funding; systematic review |  |
|  |  | registration number with registry name. |  |
| **INTRODUCTION** |  |  |  |
|  |  |  |  |
| Rationale | 3 | Describe the rationale for the review in the context of what is | 3-4 |
|  |  | already known*, including mention of why a network meta-* |  |
|  |  | *analysis has been conducted.* |  |
| Objectives | 4 | Provide an explicit statement of questions being addressed, | 4 |
|  |  | with reference to participants, interventions, comparisons, |  |
|  |  | outcomes, and study design (PICOS). |  |
| **METHODS** |  |  |  |
|  |  |  |  |
| Protocol and | 5 | Indicate whether a review protocol exists and if and where it | 4-5 |
| registration |  | can be accessed (e.g., Web address); and, if available, provide |  |
|  |  | registration information, including registration number. |  |
| Eligibility criteria | 6 | Specify study characteristics (e.g., PICOS, length of follow-up) | 5-6 |
|  |  | and report characteristics (e.g., years considered, language, |  |
|  |  | publication status) used as criteria for eligibility, giving |  |
|  |  | rationale. *Clearly describe eligible treatments included in the* |  |
|  |  | *treatment network, and note whether any have been clustered* |  |
|  |  | *or merged into the same node (with justification).* |  |
| Information sources | 7 | Describe all information sources (e.g., databases with dates of | 5 |
|  |  | coverage, contact with study authors to identify additional |  |
|  |  | studies) in the search and date last searched. |  |
| Search | 8 | Present full electronic search strategy for at least one database, | 5 |
|  |  | including any limits used, such that it could be repeated. |  |
| Study selection | 9 | State the process for selecting studies (i.e., screening, | 5-6 |
|  |  | eligibility, included in systematic review, and, if applicable, |  |
|  |  |  |  |

|  |  | included in the meta-analysis). |  |  |
| --- | --- | --- | --- | --- |
|  |  |  |  |  |
| Data collection | 10 | Describe method of data extraction from reports (e.g., piloted |  | 6 |
| process |  | forms, independently, in duplicate) and any processes for |  |  |
|  |  | obtaining and confirming data from investigators. |  |  |
| Data items | 11 | List and define all variables for which data were sought (e.g., |  | 5 |
|  |  | PICOS, funding sources) and any assumptions and |  |  |
|  |  | simplifications made. |  |  |
| **Geometry of the** | **S1** | Describe methods used to explore the geometry of the |  | 7 |
| **network** |  | treatment network under study and potential biases related to it. |  |  |
|  |  | This should include how the evidence base has been |  |  |
|  |  | graphically summarized for presentation, and what |  |  |
|  |  | characteristics were compiled and used to describe the evidence |  |  |
|  |  | base to readers. |  |  |
| Risk of bias within | 12 | Describe methods used for assessing risk of bias of individual |  | 6-7 |
| individual studies |  | studies (including specification of whether this was done at the |  |  |
|  |  | study or outcome level), and how this information is to be used |  |  |
|  |  | in any data synthesis. |  |  |
| Summary measures | 13 | State the principal summary measures (e.g., risk ratio, |  | 7 |
|  |  | difference in means). *Also describe the use of additional* |  |  |
|  |  | *summary measures assessed, such as treatment rankings and* |  |  |
|  |  | *surface under the cumulative ranking curve (SUCRA) values,* |  |  |
|  |  | *as well as modified approaches used to present summary* |  |  |
|  |  | *findings from meta-analyses.* |  |  |
| Planned methods of | 14 | Describe the methods of handling data and combining results of |  | 7-8 |
| analysis |  | studies for each network meta-analysis. This should include, |  |  |
|  |  | but not be limited to: |  |  |
|  |  | • *Handling of multi-arm trials;* |  |  |
|  |  | • *Selection of variance structure;* |  |  |
|  |  | • *Selection of prior distributions in Bayesian analyses;* |  |  |
|  |  | *and* |  |  |
|  |  | •*Assessment of model fit.* |  |  |
| **Assessment of** | **S2** | Describe the statistical methods used to evaluate the agreement |  | 8 |
| **Inconsistency** |  | of direct and indirect evidence in the treatment network(s) |  |  |
|  |  | studied. Describe efforts taken to address its presence when |  |  |
|  |  | found. |  |  |
| Risk of bias across | 15 | Specify any assessment of risk of bias that may affect the | 7 | 6 |
| studies |  | cumulative evidence (e.g., publication bias, selective reporting |  |  |
|  |  | within studies). |  |  |
| Additional analyses | 16 | Describe methods of additional analyses if done, indicating |  | - |
|  |  | which were pre-specified. This may include, but not be limited |  |  |
|  |  | to, the following: |  |  |

• Sensitivity or subgroup analyses; • Meta-regression analyses;

• *Alternative formulations of the treatment network; and* • *Use of alternative prior distributions for Bayesian*

*analyses (if applicable).*

| **RESULTS†** |  |  |  |
| --- | --- | --- | --- |
|  |  |  |  |
| Study selection | 17 | Give numbers of studies screened, assessed for eligibility, and | 8, Fig 1 |
|  |  | included in the review, with reasons for exclusions at each |  |
|  |  | stage, ideally with a flow diagram. |  |
| **Presentation of** | **S3** | Provide a network graph of the included studies to enable | Fig 2 |
| **network structure** |  | visualization of the geometry of the treatment network. |  |
| **Summary of** | **S4** | Provide a brief overview of characteristics of the treatment | 8-9 |
| **network geometry** |  | network. This may include commentary on the abundance of |  |
|  |  | trials and randomized patients for the different interventions |  |
|  |  | and pairwise comparisons in the network, gaps of evidence in |  |
|  |  | the treatment network, and potential biases reflected by the |  |
|  |  | network structure. |  |
| Study | 18 | For each study, present characteristics for which data were | 9-10,Table 1 |
| characteristics |  | extracted (e.g., study size, PICOS, follow-up period) and |  |
|  |  | provide the citations. |  |
| Risk of bias within | 19 | Present data on risk of bias of each study and, if available, any | 13-14 |
| studies |  | outcome level assessment. |  |
|  |  |  |  |
| Results of | 20 | For all outcomes considered (benefits or harms), present, for | 10, S1 Table |
| individual studies |  | each study: 1) simple summary data for each intervention |  |
|  |  | group, and 2) effect estimates and confidence intervals. |  |
|  |  | *Modified approaches may be needed to deal with information* |  |
|  |  | *from larger networks.* |  |
| Synthesis of results | 21 | Present results of each meta-analysis done, including | 11-13, Table 2-3 |
|  |  | confidence/credible intervals. *In larger networks, authors may* |  |
|  |  | *focus on comparisons versus a particular comparator (e.g.* |  |
|  |  | *placebo or standard care), with full findings presented in an* |  |
|  |  | *appendix. League tables and forest plots may be considered to* |  |
|  |  | *summarize pairwise comparisons.* If additional summary |  |
|  |  | measures were explored (such as treatment rankings), these |  |
|  |  | should also be presented. |  |
| **Exploration for** | **S5** | Describe results from investigations of inconsistency. This may | 13 |
| **inconsistency** |  | include such information as measures of model fit to compare |  |
|  |  | consistency and inconsistency models, *P* values from statistical |  |
|  |  | tests, or summary of inconsistency estimates from different |  |
|  |  | parts of the treatment network. |  |
| Risk of bias across | 22 | Present results of any assessment of risk of bias across studies | 13-14 |
| studies |  | for the evidence base being studied. |  |
|  |  |  |  |
| Results of | 23 | Give results of additional analyses, if done (e.g., sensitivity or | - |
| additional analyses |  | subgroup analyses, meta-regression analyses*, alternative* |  |
|  |  | *network geometries studied, alternative choice of prior* |  |
|  |  | *distributions for Bayesian analyses,* and so forth). |  |
| **DISCUSSION** |  |  |  |
|  |  |  |  |
| Summary of | 24 | Summarize the main findings, including the strength of | 14 |
| evidence |  | evidence for each main outcome; consider their relevance to |  |
|  |  | key groups (e.g., healthcare providers, users, and policy- |  |
|  |  | makers). |  |
| Limitations | 25 | Discuss limitations at study and outcome level (e.g., risk of | 15 |
|  |  | bias), and at review level (e.g., incomplete retrieval of |  |
|  |  | identified research, reporting bias). *Comment on the validity of* |  |
|  |  | *the assumptions, such as transitivity and consistency. Comment* |  |

|  |  | *on any concerns regarding network geometry (e.g., avoidance* |  |  |
| --- | --- | --- | --- | --- |
|  |  | *of certain comparisons).* |  |  |
|  |  |  |  |  |
| Conclusions | 26 | Provide a general interpretation of the results in the context of |  | 15-16 |
|  |  | other evidence, and implications for future research. |  |  |
|  |  |  |  |  |
| **FUNDING** |  |  |  |  |
| Funding | 27 | Describe sources of funding for the systematic review and other |  | - |
|  |  | support (e.g., supply of data); role of funders for the systematic |  |  |
|  |  | review. This should also include information regarding whether |  |  |
|  |  | funding has been received from manufacturers of treatments in |  |  |

the network and/or whether some of the authors are content experts with professional conflicts of interest that could affect use of treatments in the network.

PICOS = population, intervention, comparators, outcomes, study design.

* Text in italics indicates wording specific to reporting of network meta-analyses that has been added to guidance from the PRISMA statement.

† Authors may wish to plan for use of appendices to present all relevant information in full detail for items in this section.
