# Supplementary material for: Antithrombotic therapy strategies for atrial fibrillation patients undergoing percutaneous coronary intervention: A systematic review and network meta-analysis
Source: PLoS One. 2017 Oct 12;12(10):e0186449. doi: 10.1371/journal.pone.0186449 (PMC5638551; doi:10.1371/journal.pone.0186449)
Supplement: S1 Table — Abbreviations: MACCEs, main adverse cardiac and cerebrovascular events; DAPT, dual-antiplatelet therapy; Riva15 + P2Y12, rivaroxaban 15 mg/d plus P2Y12 inhibitor; Riva2.5 + P2Y12, rivaroxaban 2.5 mg bid plus P2Y12 inhibitor; TT, triple-antiplatelet therapy; VKA, vitamin K antagonist; SAPT, single antiplatelet therapy. (DOCX) [file pone.0186449.s002.docx]

| Riva2.5+DAPT | Total patients (n) |  |  |  |  |  |  |  |  |  |  |  |  |  | 706 |  |
| --- | --- | --- | --- | --- | --- | --- | --- | --- | --- | --- | --- | --- | --- | --- | --- | --- |
|  | Major bleeding (%) |  |  |  |  |  |  |  |  |  |  |  |  |  | 1.9 |  |
|  | Major bleeding (n) |  |  |  |  |  |  |  |  |  |  |  |  |  | 12 |  |
|  | MACCEs (%) |  |  |  |  |  |  |  |  |  |  |  |  |  | 21.7 |  |
|  | MACCEs (n) |  |  |  |  |  |  |  |  |  |  |  |  |  | 143 |  |
| Riva15+P2Y_12_ | Total patients (n) |  |  |  |  |  |  |  |  |  |  |  |  | 61 | 696 |  |
|  | Major bleeding (%) |  |  |  |  |  |  |  |  |  |  |  |  | 9.8 | 2.1 |  |
|  | Major bleeding (n) |  |  |  |  |  |  |  |  |  |  |  |  | 6 | 14 |  |
|  | MACCEs (%) |  |  |  |  |  |  |  |  |  |  |  |  | 13.1 | 21.4 |  |
|  | MACCEs (n) |  |  |  |  |  |  |  |  |  |  |  |  | 8 | 136 |  |
| VKA+SAPT | Total patients (n) |  | 279 |  |  | 51 | 73 |  |  |  |  |  |  |  |  | 31 |
|  | Major bleeding (%) |  | 3.2 |  |  | 9.8 | 6.8 |  |  |  |  |  |  |  |  | 6.45 |
|  | Major bleeding (n) |  | 9 |  |  | 5 | 5 |  |  |  |  |  |  |  |  | 2 |
|  | MACCEs (%) |  | 11.1 |  |  | 37.3 | 17.8 |  |  |  |  |  |  |  |  | 12.9 |
|  | MACCEs (n) |  | 31 |  |  | 19 | 13 |  |  |  |  |  |  |  |  | 4 |
| DAPT | Total patients (n) | 103 |  | 60 | 1200 | 67 | 162 | 166 | 236 | 3589 | 488 | 266 | 130 |  |  | 19 |
|  | Major bleeding (%) | 1.94 |  | 6.9 | 11.9 | 4.5 | 12.3 | 0.6 | 4.6 | 11.0 | 11.5 | 2.3 | 2.4 |  |  | 5.3 |
|  | Major bleeding (n) | 2 |  | 4 | 154 | 3 | 33 | 6 | 11 | 395 | 56 | 35 | 21 |  |  | 1 |
|  | MACCEs (%) | 15.5 |  | 3.3 | 20.7 | 29.9 | 20.4 | 19.3 | 17.8 | 32.7 | 20.1 | 18.4 | 21.5 |  |  | 21.1 |
|  | MACCEs (n) | 2 |  | 4 | 143 | 3 | 20 | 1 | 11 | 395 | 56 | 6 | 3 |  |  | 1 |
| TT | Total patients (n) | 14 | 284 | 44 | 448 | 28 | 679 | 37 | 131 | 1370 | 371 | 319 | 159 | 92 | 697 | 48 |
|  | Major bleeding (%) | 0 | 5.6 | 11.1 | 14.4 | 21.4 | 10.2 | 2.7 | 16.7 | 17.6 | 6.4 | 7.5 | 11.7 | 6.5 | 3.3 | 8.3 |
|  | Major bleeding (n) | 0 | 16 | 5 | 64 | 6 | 69 | 1 | 22 | 241 | 24 | 24 | 19 | 6 | 20 | 4 |
|  | MACCEs (%) | 7.14 | 17.6 | 11.4 | 19.6 | 25.0 | 21.6 | 2.7 | 22.1 | 32.6 | 17.0 | 15.4 | 18.2 | 25.0 | 29.3 | 27.1 |
|  | MACCEs (n) | 1 | 50 | 5 | 88 | 7 | 147 | 1 | 29 | 447 | 63 | 49 | 29 | 23 | 175 | 13 |
| Trial/country | | Germany | WOEST | Poland | CRUSADE | Japan | AFCAS | Korea Suh | Korea Kang | ACTION | AVIATOR | Spain | Triple Elderly | ROCKET-AF | PIONEER AF-PCI | Italy |
| No. | | 1 | 2 | 3 | 4 | 5 | 6 | 7 | 8 | 9 | 10 | 11 | 12 | 13 | 14 | 15 |
