# Supplementary material for: Antithrombotic therapy strategies for atrial fibrillation patients undergoing percutaneous coronary intervention: A systematic review and network meta-analysis
Source: PLoS One. 2017 Oct 12;12(10):e0186449. doi: 10.1371/journal.pone.0186449 (PMC5638551; doi:10.1371/journal.pone.0186449)
Supplement: S2 Table — Abbreviations: RCT, randomized clinical trial. (DOCX) [file pone.0186449.s003.docx]

| Trial | Author | Year | Randomization | Double blind | Withdrawals and dropouts | Jadad score |
| --- | --- | --- | --- | --- | --- | --- |
| WOEST | Willem J M Dewilde, et al | 2013 | 2 | 0 | 1 | 3 |
| ROCKET AF | Matthew W. Sherwood, et al | 2016 | 2 | 2 | 1 | 5 |
| PIONEER AF-PCI | Gibson CM, et al | 2016 | 2 | 0 | 1 | 3 |
