# Supplementary material for: Antithrombotic therapy strategies for atrial fibrillation patients undergoing percutaneous coronary intervention: A systematic review and network meta-analysis
Source: PLoS One. 2017 Oct 12;12(10):e0186449. doi: 10.1371/journal.pone.0186449 (PMC5638551; doi:10.1371/journal.pone.0186449)
Supplement: S3 Table — (DOCX) [file pone.0186449.s004.docx]

| Trial/country | Author | Year | Selection | Comparability | Outcome | NOS score |
| --- | --- | --- | --- | --- | --- | --- |
| Germany | Lars Maegdefessel, et al | 2008 | 4 | 2 | 2 | 8 |
| Poland | Magdalena Dąbrowska, et al | 2013 | 4 | 1 | 2 | 7 |
| CRUSADE registry | Emil L. Fosbol, et al | 2013 | 4 | 1 | 2 | 7 |
| Japan | Hideki Kawai, et al | 2014 | 4 | 1 | 2 | 7 |
| AFCAS | Andrea Rubboli, et al | 2014 | 4 | 1 | 2 | 7 |
| Korea | Soon Yong Suh, et al | 2014 | 4 | 2 | 2 | 8 |
| Korea | Dong Oh Kang, et al | 2015 | 4 | 2 | 2 | 8 |
| ACTION Registry–GWTG | Connie N. Hess, et al | 2015 | 4 | 1 | 2 | 7 |
| AVIATOR | Marco G. Mennuni, et al | 2015 | 4 | 1 | 2 | 7 |
| Spain | Antonia Sambola, et al | 2016 | 4 | 1 | 2 | 7 |
| Triple Therapy in Elderly Patients | Antonia Sambola, et al | 2016 | 4 | 1 | 2 | 7 |
| Italy | Renato De Vecchis, et al | 2016 | 4 | 0 | 2 | 6 |

NOS, Newcastle-Ottawa Scale.
